# Layer-Dependent Magnetic Domains in Atomically Thin Fe_5_GeTe_2_

**DOI:** 10.1021/acsnano.2c01948

**Published:** 2022-07-08

**Authors:** Ryuji Fujita, Pedram Bassirian, Zhengxian Li, Yanfeng Guo, Mohamad A. Mawass, Florian Kronast, Gerrit van der Laan, Thorsten Hesjedal

**Affiliations:** † Clarendon Laboratory, Department of Physics, University of Oxford, Parks Road, Oxford, OX1 3PU, United Kingdom; ‡ Max Planck Institute of Microstructure Physics, Weinberg 2, 06120 Halle, Germany; ¶ School of Physical Science and Technology, ShanghaiTech University, Shanghai 201210, China; § Helmholtz-Zentrum Berlin für Materialien und Energie, Albert-Einstein-Strasse 15, 12489 Berlin, Germany; ∥ Diamond Light Source, Harwell Science and Innovation Campus, Didcot, OX11 0DE, United Kingdom

**Keywords:** two-dimensional material, Fe_5_GeTe_2_, Fe_3_GeTe_2_, magnetic materials, van der Waals materials

## Abstract

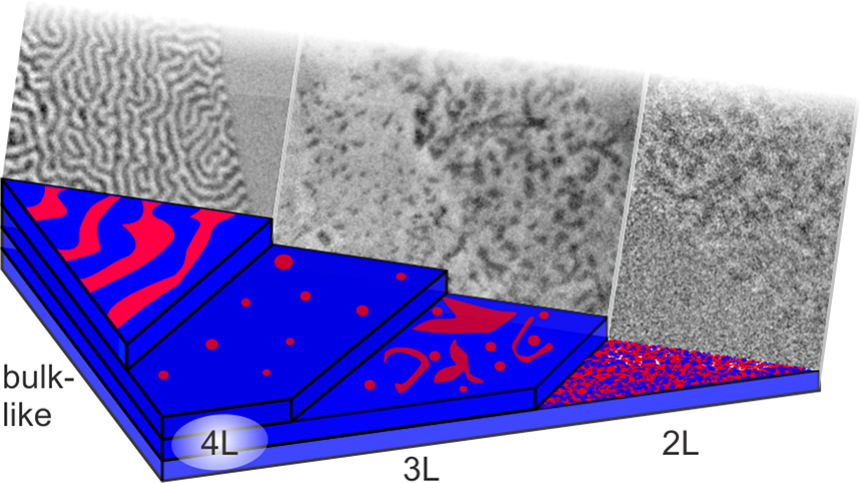

Magnetic domain formation in two-dimensional (2D) materials gives perspectives into the fundamental origins of 2D magnetism and also motivates the development of advanced spintronics devices. However, the characterization of magnetic domains in atomically thin van der Waals (vdW) flakes remains challenging. Here, we employ X-ray photoemission electron microscopy (XPEEM) to perform layer-resolved imaging of the domain structures in the itinerant vdW ferromagnet Fe_5_GeTe_2_ which shows near room temperature bulk ferromagnetism and a weak perpendicular magnetic anisotropy (PMA). In the bulk limit, we observe the well-known labyrinth-type domains. Thinner flakes, on the other hand, are characterized by increasingly fragmented domains. While PMA is a characteristic property of Fe_5_GeTe_2_, we observe a spin-reorientation transition with the spins canting in-plane for flakes thinner than six layers. Notably, a bubble phase emerges in four-layer flakes. This thickness dependence, which clearly deviates from the single-domain behavior observed in other 2D magnetic materials, demonstrates the exciting prospect of stabilizing complex spin textures in 2D vdW magnets at relatively high temperatures.

## Introduction

The celebrated discovery of atomically thin graphene^1^ in 2004 has ignited the search for other 2D materials with profoundly distinct properties from their bulk counterparts. This trend has continued with the exfoliation and magnetic characterization of atomically thin CrI_3_^2^ and CrGeTe_3_,^3^ in which magnetic order has been reported down to the monolayer and bilayer, respectively. Since then, a large and varied assortment of 2D ferromagnets (FMs) and antiferromagnets (AFMs) has been discovered.^4−7^

Moreover, exotic magnetic textures, such as skyrmions, have been found in 2D FM-transition metal dichalcogenide (TMDC)^8^ and 2D FM-FM^9^ heterostructures, as well as oxidized flakes of 2D FMs.^10^ In such cases, the antisymmetric exchange interaction, the so-called Dzyalozhinskii–Moriya interaction (DMI), plays a key role in the skyrmion stabilization.^11^ In principle, a variety of interactions, such as the dipolar interaction^12^ and ferroelectric coupling,^13^ lend multiple degrees of freedom to stabilize and move skyrmions in 2D magnets.

Nevertheless, the characterization of skyrmions, and, more generally, magnetic domains in atomically thin magnets, is challenging, due to the lack of lateral resolution and depth sensitivity in the 2D regime. Due to such constraints, surface-sensitive microscopy techniques including magneto-optical Kerr effect (MOKE) microscopy^2,3^ and nitrogen-vacancy (NV) center magnetometry, as well as magnetic force microscopy (MFM),^4^ have been employed to directly image magnetic domains in atomically thin magnets. Indeed, a recent demonstration of moiré magnetism in twisted CrI_3_ bilayers illustrates the discovery of low-dimensional magnetic orders afforded by the submicron resolution and nonperturbative nature of scanning NV magnetometry,^14^ and motivates the real-space imaging of other exfoliated vdW magnets.

A rather special compound out of the magnetic vdW materials family is Fe_5_GeTe_2_, which is closely related to the widely investigated Fe_3_GeTe_2_. In magnetic transition metal halides (Cr*X*_3_, X = I, Cl, Br, and NiI_2_), large magnetoresistance values have been observed in vdW magnetic tunnel junctions.^6,7,15,16^ In principle, the electronic itineracy in Fe_3,4,5_GeTe_2_^17,18^ allows for carrier-mediated, magnetoelectric coupling. Furthermore, bulk Fe_5_GeTe_2_ boasts a high *T*_C_ of 270–363 K,^19−21^ despite its weak perpendicular magnetic anisotropy (PMA) and coercivity (*H*_*C*_ = 50 Oe at 2 K)^19^ compared to that of Fe_3_GeTe_2_ (*H*_*C*_ = 4000 Oe at 55 K).^4^

To understand the itinerant high-*T*_C_ ferromagnetism in bulk Fe_5_GeTe_2_, we elucidate several potential origins by considering the role of electron itineracy and delocalized Te ligands in mediating the ferromagnetic coupling. In fact in Fe_5_GeTe_2_, the Te 5*p* state has been found to have a finite net spin polarization.^18^ In other words, the Te site plays a direct role in mediating the coupling between the Fe 3*d* sites, while, in insulating CrGeTe_3_, the Te 5*p* states are located well below the Fermi level (*E*_F_) and indirectly mediate the coupling between Cr *t*_2*g*_ sites via ferromagnetic superexchange.^22^ In the case of Fe_5_GeTe_2_, strong hybridization of Fe 3*d* and Te 5*p* states near *E*_F_ would account for the finite spin moment on the Te site and the itinerant ferromagnetism in general, which has direct consequences for the magnetic crystalline and exchange anisotropies, which could lead to exotic magnetic ground states.^23^ The direct evaluation of the critical exponents of bulk Fe_5_GeTe_2_^24^ and Fe_3_GeTe_2_^25^ reveal simultaneous 3D Heisenberg and 3D Ising-type couplings, while CrSiTe_3_ follows a 2D Ising behavior even in the bulk limit.^26^ The apparent 3D magnetic exchange in bulk Fe_5_GeTe_2_ and the weak PMA motivates the characterization of its low-dimensional magnetic behavior.

**Figure 1:**
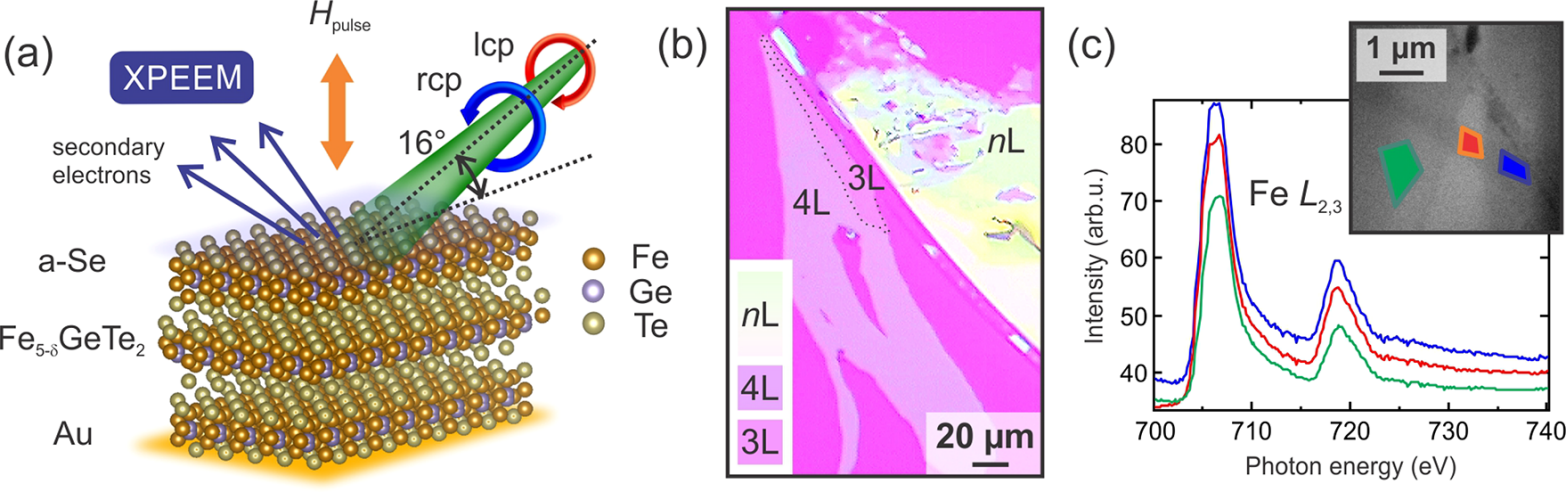
XPEEM setup and exfoliated Fe_**5**_GeTe_2_ flake. (a) Experimental XPEEM setup and layered Fe_5_GeTe_2_ crystal structure on an Au underlayer. The sample was capped with an amorphous Se (a-Se) layer. (b) Optical micrograph showing exfoliated flake with trilayer (3L, marked by a dotted line), four-layer (4L), and bulk-like (*n*L) regions. The thicknesses have been cross-calibrated with atomic force microscopy (Figure S2) and via the intensity of the *L*_3_ absorption peak. (c) Spatial XAS scan of an ultrathin mono- and bilayer area of the sample. The spatial integration of the XAS spectra over the three colored areas (see XPEEM image in inset) reveals metallic Fe *L*_3_ and *L*_2_ edges, indicating the absence of oxidation (see Figure S3 for the spectrum of oxidized Fe_5_GeTe_2_).

In this work, we employ X-ray photoemission electron microscopy (XPEEM) to image ferromagnetic domains in atomically thin and bulk Fe_5_GeTe_2_ as a function of thickness (Figure 1). In the bulk limit, the established labyrinth-type ferromagnetic domains are observed, while, in four-layer (4L) Fe_5_GeTe_2_, magnetic bubbles appear among a largely single-domain state. A multidomain state is observed for thicker and thinner flakes, while bilayers (2L) and monolayers (1L) show a highly fragmented domain state. The domain patterns in Fe_5_GeTe_2_ depart from the more commonly observed single domains observed in other few-layer vdW magnets, and we ascribe this behavior to a reduction in the PMA as the layers become thinner, as evidenced by a spin reorientation transition observed below 6Ls. Moreover, we determined the *T*_C_ in 1L flakes to be 120–150 K. Such a reduction in *T*_C_ originates from the competing magnon dispersion at finite temperature^27^ and has been observed in all other 2D ferromagnetic materials, with the exception of VI_3_.^24^ Despite this reduction, the 1L *T*_C_ is still among the highest out of the family of magnetic vdW ferromagnets and raises the prospect of stabilizing complex magnetic orders in 2D vdW materials at relatively high temperatures.

The characterization of magnetic domains in 2D vdW materials demands a depth resolution commensurate to a single unit cell. Surface-sensitive and scanning probe microscopies are naturally well-suited for this purpose, and indeed, MFM,^4,28^ MOKE,^2,3,5,19^ and NV center magnetometry^13,14,29^ have been employed to characterize a vast array of magnetic 2D materials and their heterostructures. It is important to clarify that there is a large distribution of lateral and depth resolution scales even among the aforementioned surface-sensitive techniques. For instance, MFM has revealed magnetic domains in monolayer V-doped WSe_2_,^28^ but reports of MFM-resolved domain contrast in other atomically thin materials remain scarce, presumably due to the detrimental effect from the stray field of the magnetic tip.^30^ MOKE lacks the lateral resolution required to distinguish submicron magnetic domains due to its diffraction-limited resolution. In this sense, NV magnetometry sets itself apart from the other techniques due to its ability to resolve small stray fields from the sample, coupled with high lateral resolution.

X-ray based photoemission spectroscopies, carried out at synchrotron radiation facilities, offer a complementary approach to characterizing magnetic domains in atomically thin layers. X-ray photoemission electron microscopy (XPEEM) can be combined with circular or linear magnetic dichroism and utilizes the secondary electrons to reconstruct a spatial image of the element-specific magnetic domain structure of ferro-, ferri-, and antiferromagnetic samples.^31−35^ XPEEM overcomes the challenges related to diffraction-limited lateral resolution of optical methods, while having a probing depth of several nanometers, which makes it ideal for studying the magnetic properties of 2D materials.^36^ However, due the reliance on photoelectrons, only very small magnetic fields can be applied in XPEEM (field range between 10 and 75 mT depending upon sample thickness) and the measurements are primarily done at remanence. Owing to its combination of high spatial resolution (achieving typically 30 nm) and element-specificity, XPEEM is particularly suitable for performing layer-resolved measurements of magnetic heterostructures, such as the identification of topological objects in exchange-coupled α-Fe_2_O_3_/Co multilayers,^37^ topological insulator-ferromagnet heterostructures,^38^ and magnetic domains on curved substrates.^39^ In addition, given that circular dichroism in XPEEM is mapping the projection of the magnetic moments along the incident beam direction, both in-plane and out-of-plane magnetization components may be derived from images taken at different azimuthal angles, allowing for the construction of a full vector map of the magnetization.^40^ Furthermore, the elemental sensitivity allows for in situ chemical profiling, a particularly informative technique when searching for signs of oxidation of air-sensitive vdW compounds, and for layer-resolved studies of vdW heterostructures.

## Results and Discussion

### Bulk Magnetism

**Figure 2:**
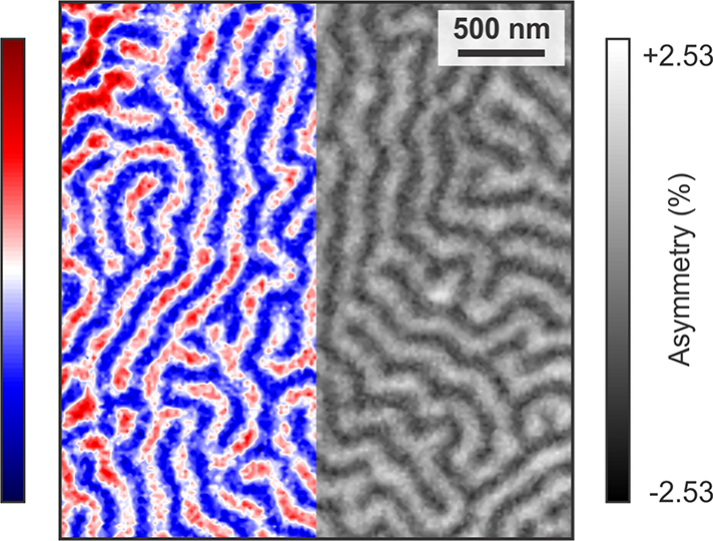
Domain structure of Fe_**5**_GeTe_2_ in the bulk limit. The XPEEM image shows maze domains, characteristic of Fe_5_GeTe_2_ in the bulk limit (*T* = 50 K). To enhance the visibility of domain walls, a color scale is used for the left-hand side of the image. Note that the domain size is smaller compared to Fe_3_GeTe_2_ (Figure S6), which is to be expected as the magnetocrystalline anisotropy is larger in Fe_3_GeTe_2_. There is almost no net magnetization at remanence (ratio of the bright to dark domain area is 53:47). The average domain width (across a stripe) is 250 nm for Fe_5_GeTe_2_ and 360 nm for Fe_3_GeTe_2_ (Figure S5), and the average domain lengths are <4 μm and >10 μm, respectively.

In bulk (>50 nm) Fe_5_GeTe_2_ flakes, we observe maze-like domain patterns (Figure 2), which are also known as stripe or labyrinth patterns. These domains have a largely out-of-plane orientation, indicating the presence of PMA and dipolar interactions. The magnetic anisotropy of Fe_5_GeTe_2_ is similar to bulk Fe_3_GeTe_2_, in which a large PMA was found,^4,5^ but different from the behavior of Fe_4_GeTe_2_, which shows a spin reorientation transition from in-plane to out-of-plane anisotropy at lower temperatures.^41^ Compared with bulk Fe_3_GeTe_2_ which also exhibits extended stripe domains, Fe_5_GeTe_2_ has a higher concentration of comparatively narrow domain walls (Figure S5), which is consistent with theoretical calculations and experimental measurements^41^ pointing to a smaller PMA in Fe_5_GeTe_2_.

### Few-Layer Magnetism

**Figure 3:**
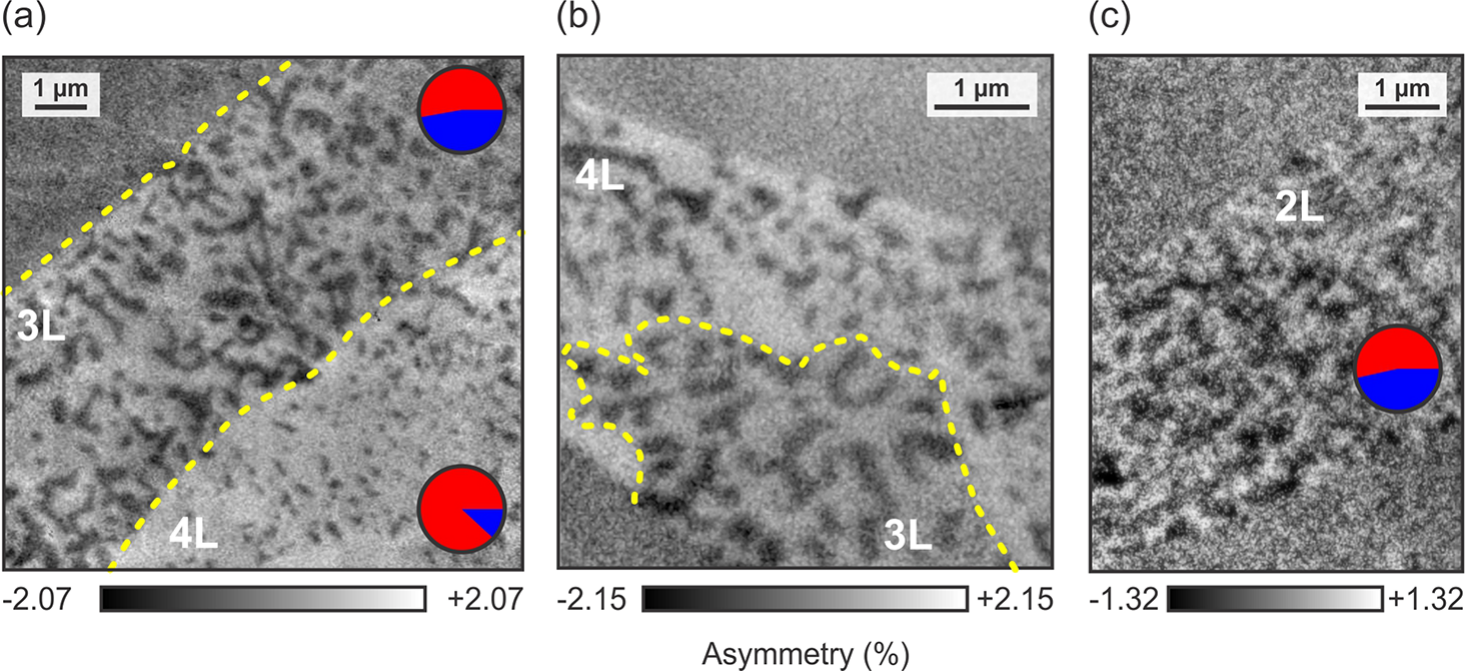
Layer-dependent magnetic domain structures. The images obtained for (a,b) 4L and 3L, and (c) 2L flakes, show a strong dependence on Fe_5_GeTe_2_ thickness (*T* = 50 K). The distribution of up (red) and down (blue) domains for each thickness are indicated in (a) and (c). The 4L area shows interdispersed isolated bubbles, which are dominating the domain contrast further away from the edge of the flake. The 3L area shows elongated domains, as well as a smaller density of interdispersed isolated bubbles. The 2L flake shows a lower magnetic signal and exhibits a highly fragmented domain state. The boundaries between the *n*L flakes are indicated by dashed yellow lines. They were obtained from the XAS maps (example shown in Figure S6). Note that the panels have different asymmetry scales.

A surprising discrete thickness dependence of the magnetic ground state begins to appear in the few-layer limit, as shown in Figure 3 for 2L, 3L, and 4L flakes. The existence of varying magnetic ground states for different thicknesses in the few-layer limit of magnetic 2D materials is unusual, and the tuning of magnetism has mostly been realized in vdW heterostructures^42,43^ or via other extrinsic means, such as gating.^44^ Note that the reported thickness dependence has been observed for layers exfoliated from different Fe_5_GeTe_2_ bulk crystals, and studied during different beamtimes. Below, we discuss several possible origins behind the thickness-dependent variation of the magnetic ground state.

In 4L regions (marked in Figure 3a,b), distributed magnetic bubbles form, which range in diameter from 300 to 500 nm, surrounded by single domains of the opposite magnetic orientation. In a binary approximation, i.e., assuming that the moments point either parallel or antiparallel to the out-of-plane easy axis,^24^ 88% of them are found to point out-of-plane. Such an asymmetry in the domain distribution is comparable to the single-domain state observed in few-layer Fe_3_GeTe_2_,^4,5^ although the formation of the small bubble domains of antiparallel orientation differentiates this 4L magnetization state from that of few-layer Fe_3_GeTe_2_. Note that the flakes were measured at remanence, without their previous exposure to external magnetic fields. We return to a more detailed discussion of these magnetic bubbles in atomically thin Fe_5_GeTe_2_ further below.

In the 3L regions, a multidomain state consisting of magnetic bubbles and stripe domains is found. Compared to 4L regions, the bubbles have larger diameters, ranging from 500 to 600 nm. The larger bubble diameters, and the generally higher concentration of domains and domain walls, could be explained by a decrease in the PMA^45−47^ or stronger long-range dipole–dipole interactions.^48^ Moreover, the stripe domains resemble bubbles interconnected with their nearest neighbors, giving the impression that a continuously varying energy term is at play, rather than a discrete change in the symmetry or stacking order.^6,49^ Here, only 52% of the domains point parallel to the easy axis in a binary approximation.

In the 2L region, highly fragmented magnetic domains, which no longer can be categorized as pointing (anti)-parallel to the easy axis, emerge. At this thickness, magnetic bubbles are no longer found, however, whether this is due to a spatial variation of the magnetic moments or spin canting could not be determined. Nevertheless, in a binary approximation, only 53% of moments point parallel, indicating the presence of energy terms comparable to the magnetic anisotropy even in the bilayer limit. The small asymmetry values, compared to the 3L, 4L, and bulk flakes, make an in-depth analysis of the bilayer domain structure challenging.

These changes in the magnetic domain structures from 4L to 3L to 2L appear to indicate a decrease in the PMA, or additional energy terms which compete with the PMA. Such behavior would contrast the single-domain, eas*y*-axis ferromagnetism observed in Fe_3_GeTe_2_^4,36,50^ and CrGeTe_3_,^3^ and the layer-dependent antiferromagnetism in CrI_3_.^2,14,29,51^ In either case, an in-plane magnetization component can be expected to be present in the thinner layers. Next, we investigate the anisotropy of the flakes in more detail.

### Magnetic Anisotropy

**Figure 4:**
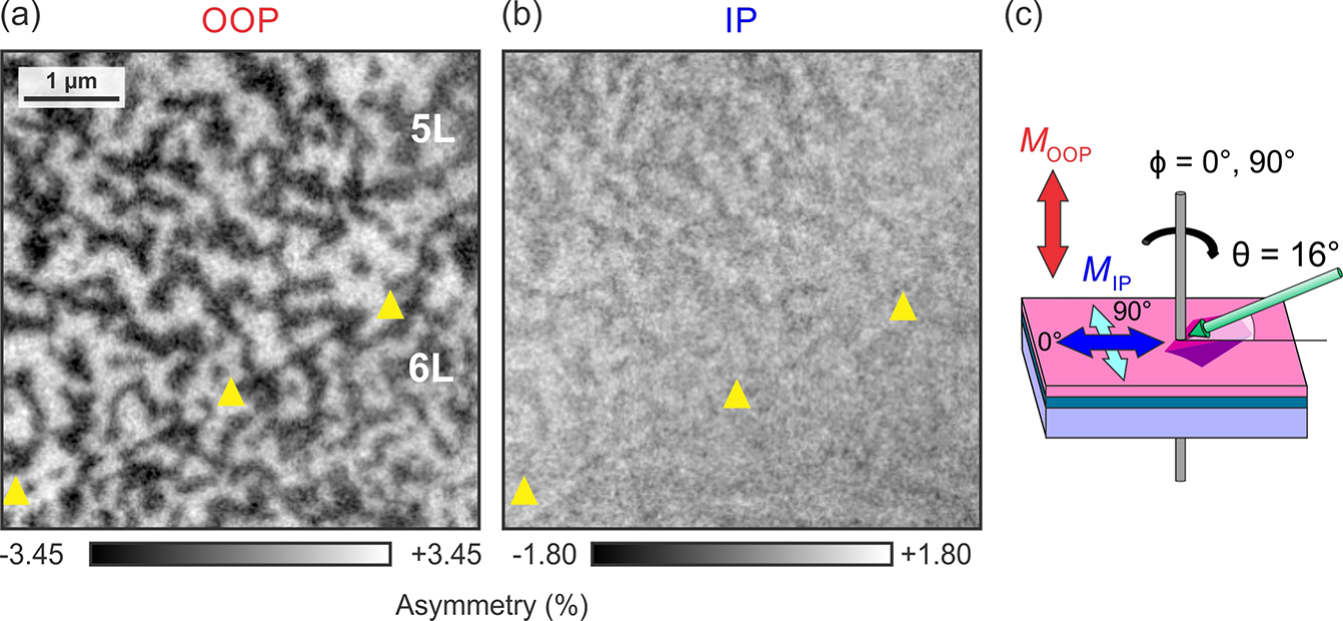
Magnetic anisotropy of Fe_**5**_GeTe_2_. (a) Out-of-plane (OOP) and (b) in-plane (IP) magnetization patterns derived from XPEEM images taken at 0° and 90° azimuths (*T* = 50 K), as shown in (c). The 5L area has a greater in-plane magnetization component compared to the 6L area. The two layers are demarcated by the yellow triangles. Note that the panels have different asymmetry scales.

In order to gain insight into the processes that determine the change of domain structure with decreasing thickness, we carried out XPEEM imaging for different azimuths (Figure 4). In XPEEM, the contrast is directly proportional to the projection of the local moments onto the incoming X-ray wavevector. Given the incidence angle of 16°, both out-of-plane and in-plane magnetization components are obtained as geometrical projections. However, to obtain the full in-plane contrast, the azimuthal angle has to be varied (typically only 0° and 90° are required). Indeed, by performing vector XPEEM imaging, we observe an in-plane spin canting at a six-layer (6L) to five-layer (5L) boundary (Figure 4), in which the 5L area possesses a larger in-plane magnetization component than the 6L area. For magnetism to be stable in two dimensions, rotational symmetry must be broken either by a magnetocrystalline anisotropy or by long-range dipole–dipole interactions.^27,48^ Compared to Fe_3_GeTe_2_, in which the PMA term becomes dominant in the few-layer limit,^4,5,44^ the 6L to 5L spin reorientation transition in Fe_5_GeTe_2_ suggests a decrease in PMA, or enhanced energy terms including dipole–dipole and exchange interaction which compete with the PMA in the atomically thin limit. For the case in which the dipole–dipole interaction is dominant, the magnetic moments lie in the in-plane direction.^48^ An additional possibility may be that the exchange interaction within the unit cell is not strictly two-dimensional, due to the complex distribution of Fe sites,^41^ meaning that, in atomically thin Fe_5_GeTe_2_, additional energy terms may influence the magnetism in addition to the PMA.

### Monolayer Magnetism

**Figure 5:**
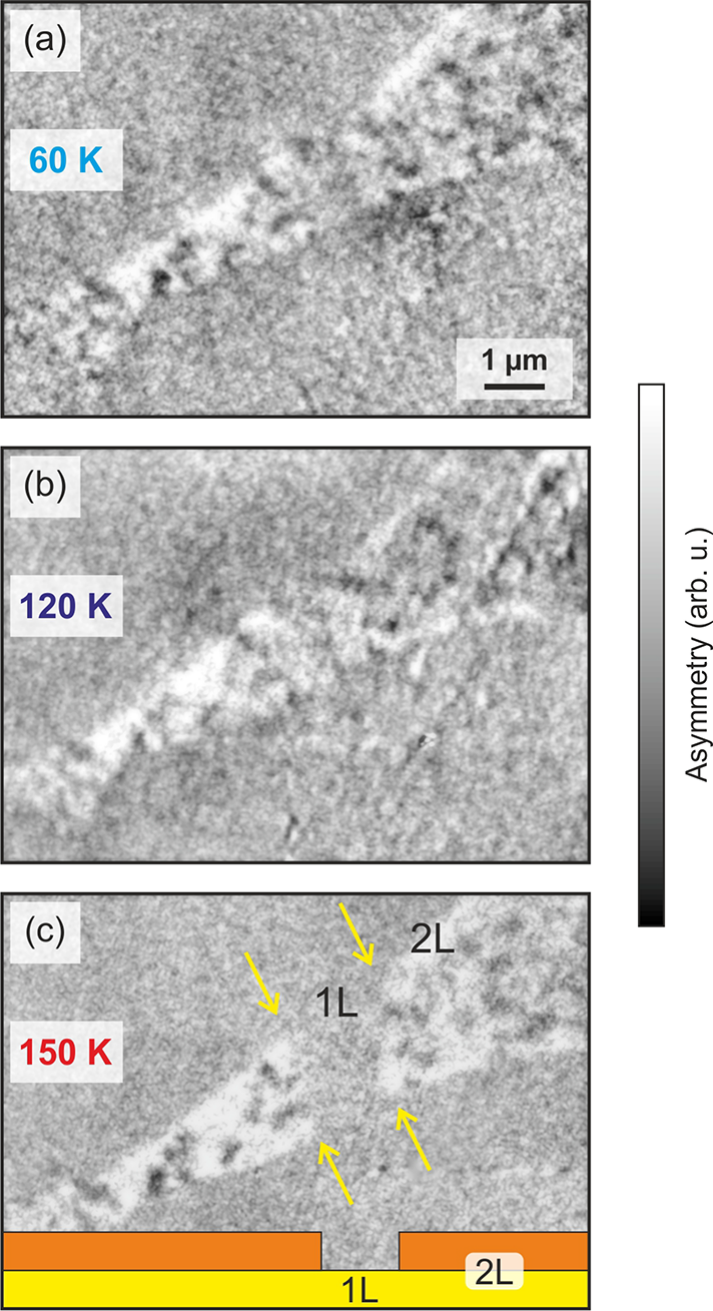
Magnetic contrast of Fe_**5**_GeTe_2_ monolayers and determination of the transition temperature. (a–c) Temperature dependent XPEEM images of bi- and monolayer Fe_5_GeTe_2_ flakes. At 60 and 120 K, the middle section of the flake, which is a monolayer (see sketch in (c)), shows clear domain contrast. The size and distribution of the domains is similar to the neighboring bilayer areas. Note that, in contrast to Fe_5_GeTe_2_, Fe_3_GeTe_2_ exhibits a single-domain state in the monolayer limit.^36^ Above 150 K, the contrast in the monolayer area has vanished, indicating a transition temperature between 120 and 150 K. The edge of the monolayer area is indicated by yellow arrows.

A magnetic phase transition, indicated by the onset of domain formation at 120–150 K (Figure 5), is observed for the 1L region surrounded by neighboring 2L regions. The 2L region to the left of the 1L exhibits a bubble-like state comparable to the four-layer ground state, while the 2L region to the right of the 1L exhibits a highly fragmented domain pattern as described above. The strong reduction of the domain size in Fe_5_GeTe_2_ for 1L likens the behavior of ultrathin transition metal PMA films in which the magnetization remains perpendicular by introducing domains, thereby reducing the shape anisotropy.^52^ The bubble-like state in the left 2L could be ascribed to finite size effects, which would induce a PMA.^53,54^ The few-layer phenomena described above occur at comparable temperatures to the widely investigated Fe_3_GeTe_2_, and at relatively higher temperatures compared to the magnetic trichalcogenides and transition metal halides. The high degree of tunability, in particular the incorporation of dopants, affords the further optimization of the transition temperature. Indeed, a *T*_C_ of 363 K has been achieved in Co-doped Fe_5_GeTe_2_.^21^

### Magnetic Bubbles

**Figure 6:**
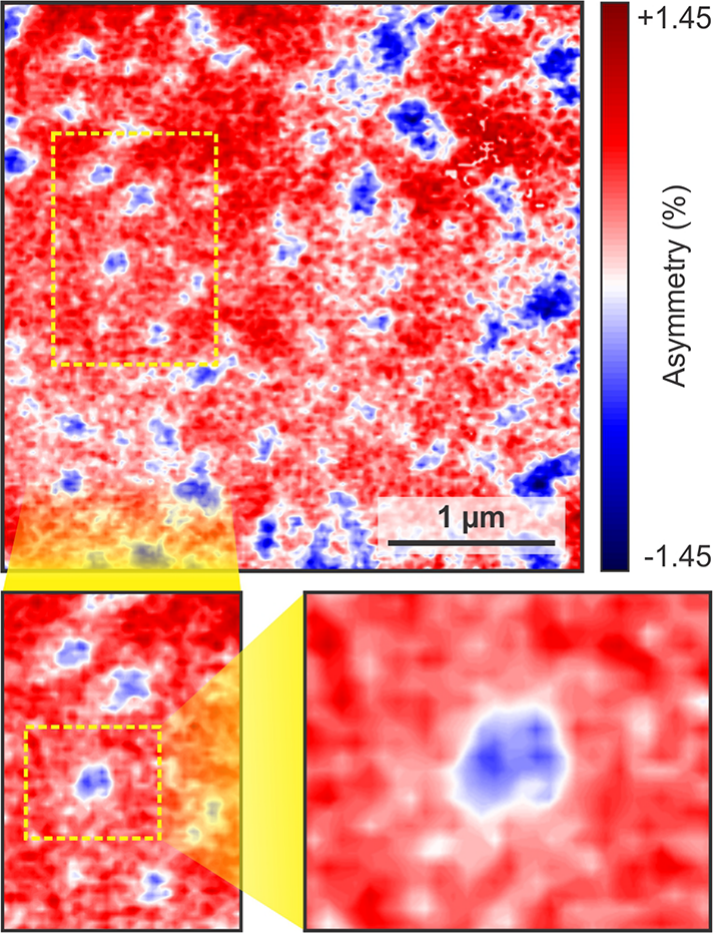
Observation of magnetic bubbles in a 4L flake. The domain structure of a 4L flake is dominated by isolated, round domains, as well as some extended features. Successive close-ups of the isolated features reveal their magnetic bubble nature. The position of the magnified areas is indicated by the respective dashed rectangles (*T* = 50 K).

As shown in Figure 3, isolated magnetic bubbles are distributed among a single majority domain in 4L flakes. In Figure 6, we take a closer look at these bubble domains. While a typical bubble only measures some 100 nm across, the transition from parallel to antiparallel magnetization occurs over a rather narrow region. Therefore, while the size is only a factor of 2 larger than topological nontrivial skyrmions in Fe_3_GeTe_2_,^55^ the transition region is untypically narrow for a skyrmion.^56^ Note, however, that topological spin textures have indeed been observed in Fe_5_GeTe_2_, including (anti)-merons,^57^ while their origin in this structurally complex magnetic material may have several possible origins stemming from disorder^58^ and additional short-range order,^19^ which could lead to the breaking of inversion symmetry and thus the emergence of the DMI. Unfortunately, resolving the details of the transition region was not possible with XPEEM, and therefore other methods, such as NV center microscopy or spin-polarized scanning tunneling microscopy, will have to be employed to shed more light on the issue.

Assuming a topologically nontrivial nature of the skyrmion bubble, apart from PMA and dipole–dipole coupling, DMI has to be present to twist the domain walls;^55^ however, the origin of any DMI for only this particular thickness would be surprising. Nevertheless, we can exclude any surface-oxide induced DMI,^10^ as the Fe *L*_2,3_ XAS consists of a single metallic peak, with no signs of a multiplet structure (Figure 1c), a clear indication that the sample is free of oxidation. Furthermore, we can exclude detrimental effects from the Se capping layer. Assuming that Se intermixing would form a sizable, nonferromagnetic FeSe_*x*_Te_1–*x*_^59−61^ layer, the magnetic contrast from Fe_5_GeTe_2_ would be largely suppressed, which is in contrast to our observation of magnetic domains from one single Fe_5_GeTe_2_ monolayer. Further, the agreement of the observed labyrinth bulk domains with the ones observed with other methods on uncapped samples^4,12,36^ suggests that Se has not altered the magnetic properties of Fe_5_GeTe_2_ either.

On the other hand, interstitial lattice defects can break local inversion symmetries and have been found to induce skyrmion formation in Fe_3_GeTe_2_.^24,62^ Furthermore, defects in CrBr_3_ have been found to pin domains, resulting in isolated magnetic bubbles.^13^ Although it is unclear why such defects would result in a thickness dependence, they cannot be excluded, due to their high prevalence and pronounced effects on the magnetism. In addition, stacking faults along the *c*-axis of exfoliated flakes^6,49^ would break the inversion symmetry between adjacent vdW layers,^19,58^ which may induce a DMI. Such a structural transition may be intrinsic to the material itself, as observed in the cases above, or may be externally induced via an interaction with the Au substrate.^63^ A close-up of a different 4L flake (Figure 6) shows a variation in the skyrmion bubble diameter of ∼300–500 nm. This variation indeed hints at a defect-induced contribution to the energy balance within the 4L flake. With 88% of the domains pointing up (Figure 3a), a large PMA contribution can be assumed.

### Micromagnetic Simulations

**Figure 7:**
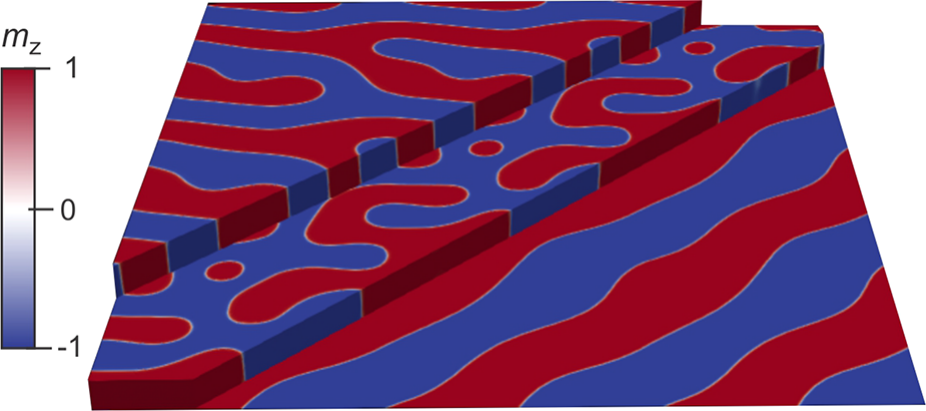
Micromagnetic simulation results showing the domain structures for flakes of different thickness at remanence. While the very thin layer is characterized by long-wavelength modulations, the thick layer shows stripe domains. In-between, at intermediate thicknesses, magnetic bubble domains emerge. Their occurrence is the result of the thickness-dependent dominance of the exchange energy over dipole–dipole coupling.

To shed more light on the possible causes of the strong thickness dependence of the magnetic domain structure, we carried out micromagnetic simulations using MuMax3.^64^ The results of the simulations for a system with three layer thicknesses, which can be characterized as thin, intermediate, and thick, are shown in Figure 7. While the very thin layer is characterized by extended domains, the thick layer shows short stripe domains, in line with the experimental findings. From these results, it can be conluded that the observed fragmented domains in the 1L and 2L limit are governed by effects which are not captured by the simulations. In-between, at intermediate thicknesses in the Goldilocks zone, bubble domains are found. Note that the results shown in Figure 7 were obtained without taking DMI into account; i.e., the observed skyrmion bubbles are large and comparable to the ones observed in multilayer systems with interfacial DMI.^65^ In this case, the cross-sectional domain walls of the bubbles have varying character; i.e., they are neither Bloch- or Néel-type, and the bubbles are therefore not topological objects. However, once a DMI term of sufficient strength is present, the stability of the bubbles increases, which is to be expected given the larger coupling energy. Further, with DMI, the helicity of the walls gets defined, as reported previously for Fe_3_GeTe_2_,^55^ and the bubbles can be characterized as skyrmions with a defined topological winding number. In this region, a topological protection can be the source of an increased stability. The previously described Goldilocks zone, in which skyrmion bubbles appear, is also present when DMI is introduced. Due to the stronger stability region, it is present for a wider range of thicknesses.

While we are not able to unambiguously determine whether the observed magnetic bubbles are topological objects, for which we would need high-resolution imaging of the detailed 3D domain structure in the transition region between core and exterior, we will discuss the strong thickness-dependence of the domain structure from a more basic standpoint. Magnetic domains usually form to reduce the magnetostatic energy of the system (demagnetization: *E*_demag_ ↓), whereby the necessary introduction of separating domain walls costs energy (anisotropy: *E*_ani_ ↑). In this bulk PMA system, the magnetic anisotropy energy density is, to first order, constant and independent of thickness. The shape anisotropy, on the other hand, which is due to dipolar interactions, increases with decreasing film thickness, forcing the magnetic moments to lie in the film plane. Such a reorientation transition is indeed observed between 5L and 6L, evidenced by the occurrence of an in-plane magnetization component (Figure 4). For magnetic bubbles to form, which have a high density of domain walls, either the energy required to form a wall has to reduce or their overall density has to be low. As can be seen in Figure 3a, the formation of bubbles in the 4L flake is tied to a quasi-single-domain state surrounding them, which reduces the overall *E*_ani_ at the cost of *E*_demag_. The 3L (Figure 3a) and 5L (Figure 4a), on the other hand, appear to have very similar, shorter stripe-like domains. This means that, at 4L, it is energetically favorable for Fe_5_GeTe_2_ to form magnetic bubbles, while, for very low thicknesses, the demagnetization energy can overcome the anisotropy energy, giving rise to a very fragmented domain state.^66^

## Conclusion

In summary, we have used XPEEM to uncover thickness-dependent magnetic ground states in exfoliated flakes of the vdW ferromagnet Fe_5_GeTe_2_. Our observation of isolated magnetic bubbles and stripes in four-layer and trilayer flakes, and a largely isotropic fragmented state in the bilayer, points to the presence of a reorientation transition driving the magnetic ordering below a thickness of five layers. Moreover, a monolayer *T*_C_ of 120–150 K demonstrates the possibility of stabilizing complex spin textures in atomically thin vdW materials at relatively high temperatures and zero-field and establishes XPEEM as a powerful method of characterizing domain structures in atomically thin magnets. We leave the origins of the magnetic bubble formation in four-layer flakes and the thickness-dependent magnetic behavior in Fe_5_GeTe_2_ as topics for future studies.

## Materials and Methods

### Bulk Crystal Growth

High-quality Fe_5_GeTe_2_ single crystals were grown by using the chemical vapor transport technique, employing iodine as the transport agent. A mixture of high purity elements including Fe, Ge, and Te with a ratio of 6:1:2 was mixed, sealed in an evacuated quartz tube, and slowly heated to 700 °C in a tubular furnace. After 7 days, the assembly was slowly cooled to room temperature. The crystallographic phase and crystal quality were examined on a Bruker D8 single crystal X-ray diffractometer with Mo Kα radiation (λ = 0.71073 Å) at 300 K.^67^ The chemical compositions and uniformity of stoichiometry were checked on several spots on the crystal by using energy dispersive spectroscopy, and the magnetic properties by superconducting quantum interference device (SQUID) magnetometry, yielding a transition temperature of 274 K.^67^ For comparison, we also investigated well-characterized Fe_3_GeTe_2_ crystals^17,68,69^ (data shown in the Supporting Information), which have a *T*_C_ of 220 K.

### Exfoliation of Thin Flakes

Atomically thin Fe_5_GeTe_2_ flakes were exfoliated via a gold-assisted method^70^ onto Si wafers with a 300  nm thick oxide layer. The flakes were exfoliated in an inert Ar glovebox with O_2_ and H_2_O concentrations below 10 ppm. The flake thicknesses were determined from their optical contrast, which was calibrated by atomic force microscopy (Figure S2). The flakes were then capped *in situ* with a thin (5 nm) Se layer to prevent oxidation, yet allowing for the transmission of photoelectrons, i.e., allowing for measurements on capped samples.

### Magnetic Domain Imaging

XPEEM measurements were conducted at the UE49/PGMa beamlime of the synchrotron radiation source BESSY II at the Helmholtz-Zentrum Berlin.^71^ Real-space X-ray absorption (XAS) and X-ray magnetic circular dichroism (XMCD) measurements at the Fe *L*_3_ edge (706.2 eV) were performed from 50 K to room temperature in zero applied field on the Se capped samples. All results shown here, apart from the temperature dependence in Figure 5, were obtained at a temperature of 50 K. The fixed angle of incidence of the incoming X-rays with respect to the sample surface was 16° (Figure 1a), which means that 28% of the sample’s out-of-plane magnetization component is projected along the X-ray propagation direction.^72^ The XMCD asymmetry is defined as (σ_–_ – σ_+_)/(σ_–_ + σ_+_), where σ_–_ and σ_+_ are the XAS signals at the maximum taken with left and right circularly polarized X-rays, respectively (lcp and rcp in Figure 1a).

### Micromagnetic Simulations

For the micromagnetic simulations in MuMax3,^64^ we used a cell size of 1 nm × 1 nm × 0.5 nm and a total of 256 × 256 × *n* cells (with *n* = 3, 68, and 140). Periodic boundary conditions were applied in the film plane. An exchange stiffness of *A*_ex_ = 1 pJ m^–1^ and a saturation magnetization of *M*_s_ = 580 kA m^–1^ were assumed. PMA was achieved by setting the out-of-plane uniaxial anisotropy constant to *K*_eff_ = 1 MJ m^–3^. The Gilbert damping constant was set to α = 0.5. Simulation results show relaxed states starting from a random spin configuration. The influence of a DMI term was investigated as well; however, the results shown in Figure 7 were obtained without it.

## Supporting Information Available

Section on sample properties and preparation and figures of Fe_5_GeTe_2_ flakes (optical micrograph), layer-dependent optical contrast of Fe_5_GeTe_2_ flakes and thickness calibration, the X-ray absorption spectrum of oxidized Fe_5_GeTe_2_, the magnetic domain structure of Fe_5_GeTe_2_ after positive and negative field pulses, and the domain structure of Fe_3_GeTe_2_ for two temperatures (PDF)
