# Supplementary material for: Layer-Dependent Magnetic Domains in Atomically Thin Fe5GeTe2
Source: ACS Nano. 2022 Jul 8;16(7):10545–53. doi: 10.1021/acsnano.2c01948 (PMC9331157; doi:10.1021/acsnano.2c01948)
Supplement: Supplementary file 1 — nn2c01948_si_001.pdf [file nn2c01948_si_001.pdf]

— Supporting Information for —

Layer-Dependent Magnetic Domains in Atomically Thin Fe_5GeTe_2

Ryuji Fujita,[†] Pedram Bassirian,[†] Zhengxian Li,[¶] Yanfeng Guo,[¶] Mohamad A. Mawass,[§] Florian Kronast,[§] Gerrit van der Laan,[‖] and Thorsten Hesjedal[*,†]

†*Clarendon Laboratory, Department of Physics, University of Oxford, Parks Road, Oxford, OX1 3PU, United Kingdom*

‡*Max Planck Institute of Microstructure Physics, Weinberg 2, 06120 Halle, Germany*

¶*School of Physical Science and Technology, ShanghaiTech University, Shanghai 201210, China*

§*Helmholtz-Zentrum Berlin für Materialien und Energie, Albert-Einstein-Strasse 15, 12489 Berlin, Germany*

‖*Diamond Light Source, Harwell Science and Innovation Campus, Didcot, OX11 0DE, United Kingdom*

E-mail: Thorsten.Hesjedal@physics.ox.ac.uk

S1. Sample properties and preparation

Bulk Fe_5GeTe_2 crystals were grown by chemical vapor transport (CVT) as described in detail in Ref.[1] A slightly Fe-rich mixture of the constituent Fe, Ge, and Te elements of 6:1:2 was mixed and sealed together with the iodine flux in a quartz tube, and brought to 700°C in a horizontal tube furnace, and kept at this temperature for seven days. After slowly cooling down to room temperature, the crystals, typically measuring $2 \times 2 \times 0.1\,\mathrm{mm}^3$, were analyzed by x-ray diffraction (XRD) to confirm the crystallographic phase and determine their quality. The stoichiometry was determined using energy-dispersive x-ray spectroscopy (EDS). Typical stoichiometries of Fe:Ge:Te = 4.6:1:2.1 were obtained by averaging the EDS data of five random sample spots.[1] The magnetic properties of the bulk crystal were determined using a superconducting quantum interference device (SQUID) magnetometer. A typical magnetic transition temperature of 270 K was determined, however, note that it is strongly depending on the precise stoichiometry which can vary across the crystalline sample.[1] As it is impractical to determine the precise stoichiometry $Fe_{5-\delta}GeTe_2$ (with a varying non-stoichiometry $\delta(x, y)$) of the individual areas investigated by x-ray photoelectron emission microscopy (XPEEM), we are referring to the crystals as Fe_5GeTe_2, i.e., as the idealized stoichiometry.

The Fe_5GeTe_2 flakes for the XPEEM measurements were obtained by exfoliation via a gold-assisted method.[2] An example of a large exfoliated area is shown in Figure S1. The process was carried out in an inert Ar glovebox with O_2 and H_2O concentrations below 10 ppm. In order to protect the flakes from exfoliation, they were capped in the glove box with a thin Se layer, which is deposited at room-temperature without heating the sample. A thickness of ∼5 nm prevents oxidation, as evidenced by the metallic (i.e., featureless) x-ray absorption spectra shown in the main text, yet allowing surface-sensitive soft x-ray spectroscopy at the Fe $L_{2,3}$ absorption edges.

In order to determine the thickness of the exfoliated Fe_5GeTe_2 flakes, we deduced a linear correlation between the thicknesses and optical contrasts. In detail, freshly exfoliated Fe_5GeTe_2 flakes were examined with optical microscopy (Figure S2(a)). Atomic force mi-

croscopy (AFM) scans (Figure S2(b)) were taken from monolayer (1L) to three layer (3L) regions as indicated in Figure S2(a). The monolayer thickness was determined to be 0.66 nm, which is consistent with XRD[1] and high-resolution transmission electron microscopy (HRTEM)[3] results. A plot of the optical contrasts as a function of layer thickness for 1L to 4L is shown in Figure S2(c).

In Figure S3, we compare the X-ray absorption spectroscopy (XAS) data across the Fe L_3 and L_2 edges of an unoxidized Fe_5GeTe_2 flake in the ultrathin limit with an example of an oxidized bulk-like flake. The clear signs of oxidation at the Fe edges are (i) a characteristic double peak structure at the L_2 edge, as well as (ii) an oxidation shoulder at the L_3 edge, representing a chemical shift to higher photon energies.[4] The data shown in the main text was collected on unoxidized samples, which do not show these typical oxidation features. Also, given that it is known from Fe_3GeTe_2 that the oxidation proceeds quickly and reaches $\sim$5 nm after even slight exposure to ambient conditions,[5] the fact that XMCD contrast can be seen for ultrathin flakes (see main text) is a strong hint that the flakes are unoxidized.

Note, however, that the XAS spectra obtained in XPEEM are usually broader than the ones obtained in, e.g., total-electron-yield mode by measuring the drain current. This is especially noticeable for very thin layers, where the signal is low, in which case the energy resolution of the monochromator is being reduced in order to increase the beam intensity. This broadening can then be easily mistaken for an oxidation shoulder.

In Figure S4, we compare the magnetic domain structure of Fe_5GeTe_2 in the bulk limit after positive and negative field pulses. As can be seen, the maze domains remain unchanged when applying magnetic fields of $\pm$66 mT.

Figure S5 shows the domain structure of Fe_3GeTe_2 for comparison. The XPEEM images, taken at two temperatures below T_C, reveal that the maze domains are unaffected by temperature changes between 60 and 100 K.

Finally, we show the correlation between Fe stoichiometry and magnetic contrast in Figure S6 by recording (b) the structural contrast along with the corresponding (c) out-

of-plane and (d) in-plane magnetic XPEEM contrast, respectively. As Fe_5GeTe_2 is prone to defect formation and non-stoichiometry, including stacking faults and interstitial lattice defects,[3,6] we find a distribution of Fe-rich and Fe-poor areas in Figure S6(b). While the magnetic contrast in areas of Fe deficiency is enhanced, for both the out-of-plane and in-plane components, it is reduced in areas with excess Fe. A more detailed study of the structural and chemical properties of exfoliated Fe_5GeTe_2 would be necessary for determining the type and the concentration of the defects, and their possible effects on magnetic properties of the layers. To facilitate a direct comparison of the in-plane and out-of-plane magnetization components of 5L and 6L areas as discussed in the main text, we selected the defect-free area marked by the dashed black rectangle in Figure S6(b).

Supplemental Figures

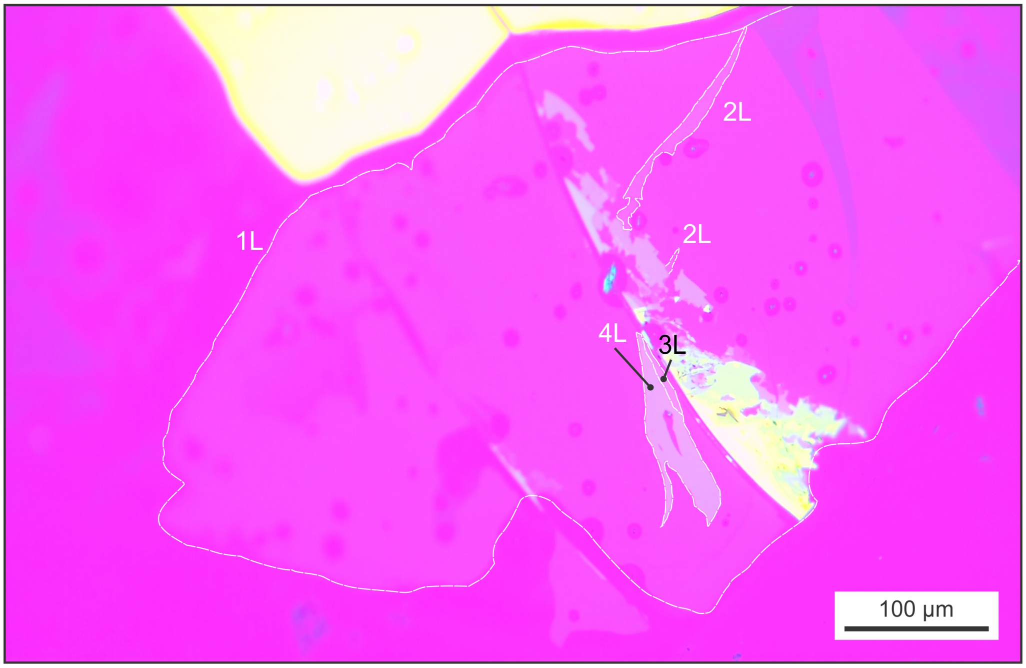

Figure S1: Optical micrograph of the Fe_5GeTe_2 flakes investigated by XPEEM.

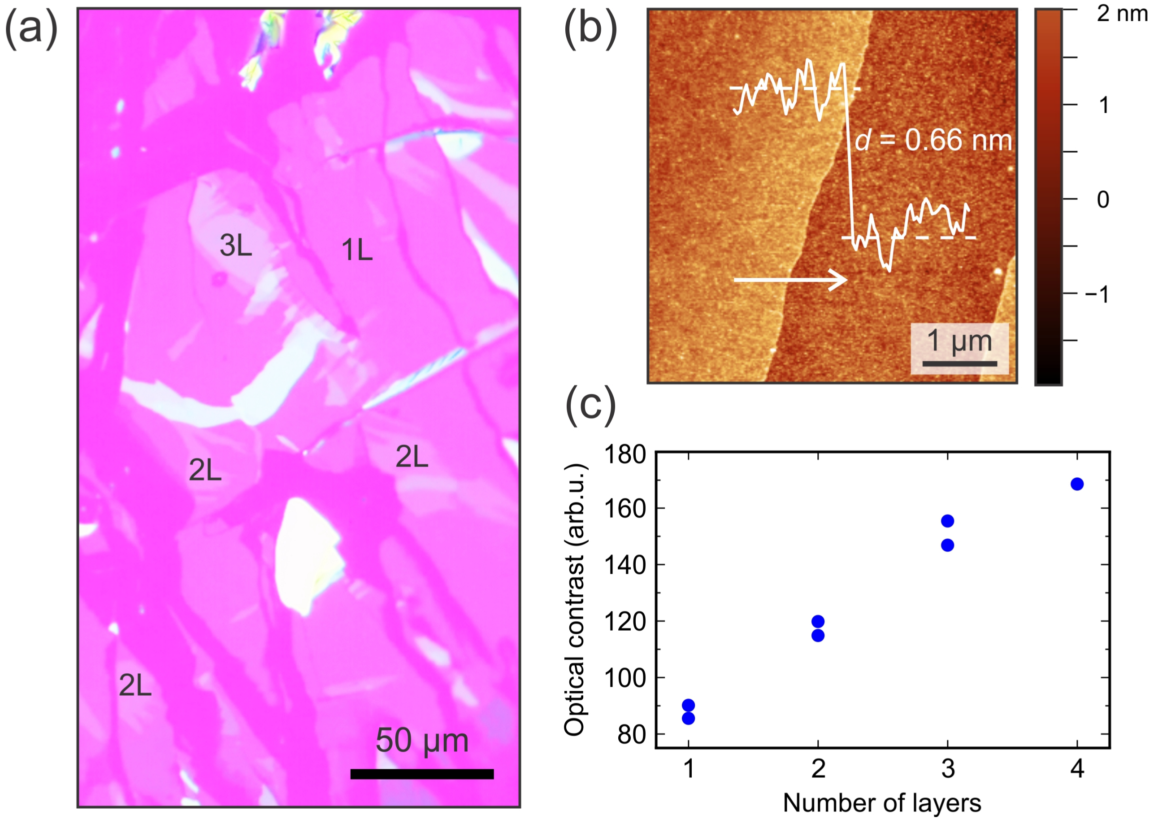

Figure S2: (a) Optical micrograph of monolayer, bilayer and trilayer Fe_5GeTe_2 flakes. (b) AFM scan showing a single monolayer thickness of 0.66 nm. (c) Layer-dependent optical contrasts for $n = 1, 2, 3$ and 4 layers.

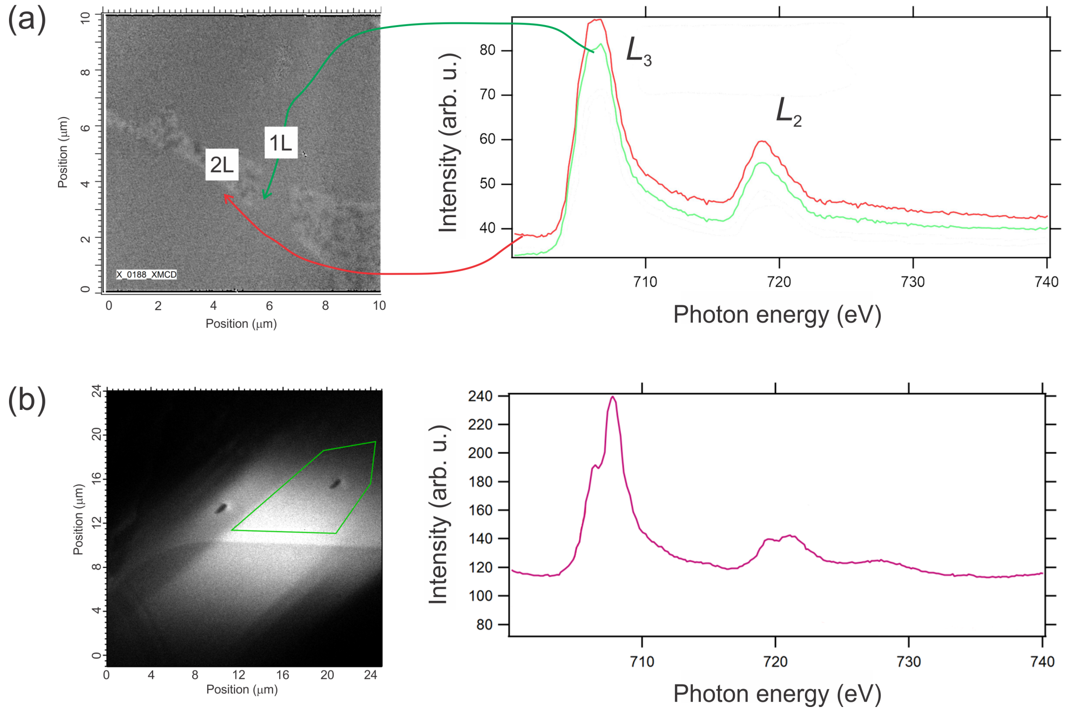

Figure S3: XAS spectra across the Fe L_3 and L_2 edges for (a) 1L and 2L areas of an unoxidized Fe_5GeTe_2 sample, and (b) a thicker area of a different, oxidized sample. Note that the oxide shows a characteristic double peak structure, which is absent on the unoxidized sample. Further, in (b), the oxidation shoulder, representing a chemical shift to higher photon energies (see, e.g., the comparison of iron compounds and oxides in Figure 9 of Ref.[4]), is very clearly visible.

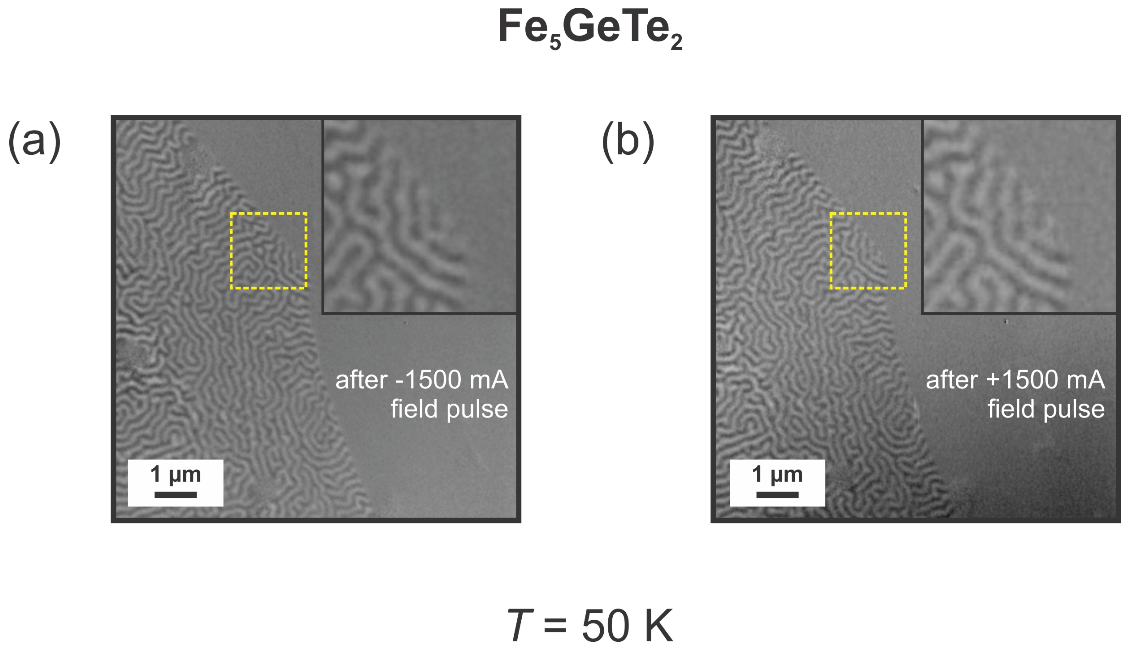

Figure S4: XPEEM images of maze domains in Fe_5GeTe_2 at 50 K. The images were taken at remanence after negative (a) and positive (b) out-of-plane magnetic field pulses of ±66 mT (magnet current ±1500 mA). As can be seen in the images, and particularly when comparing the insets of a zoomed-in area, the maze domains remain unchanged.

Fe_3GeTe_2

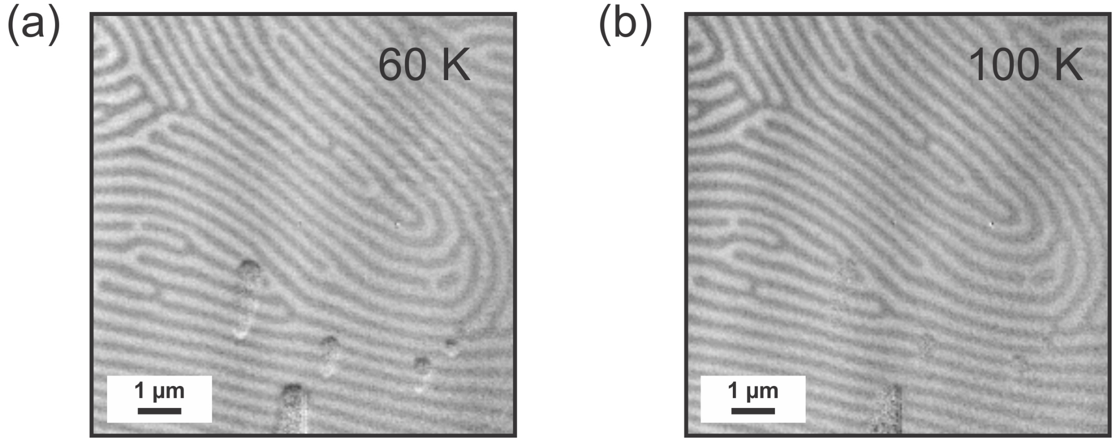

Figure S5: XPEEM images of maze domains in Fe_3GeTe_2 at 60 K (a) and 100 K (b), showing that the magnetic domain structure remains unaffected by the temperature change.

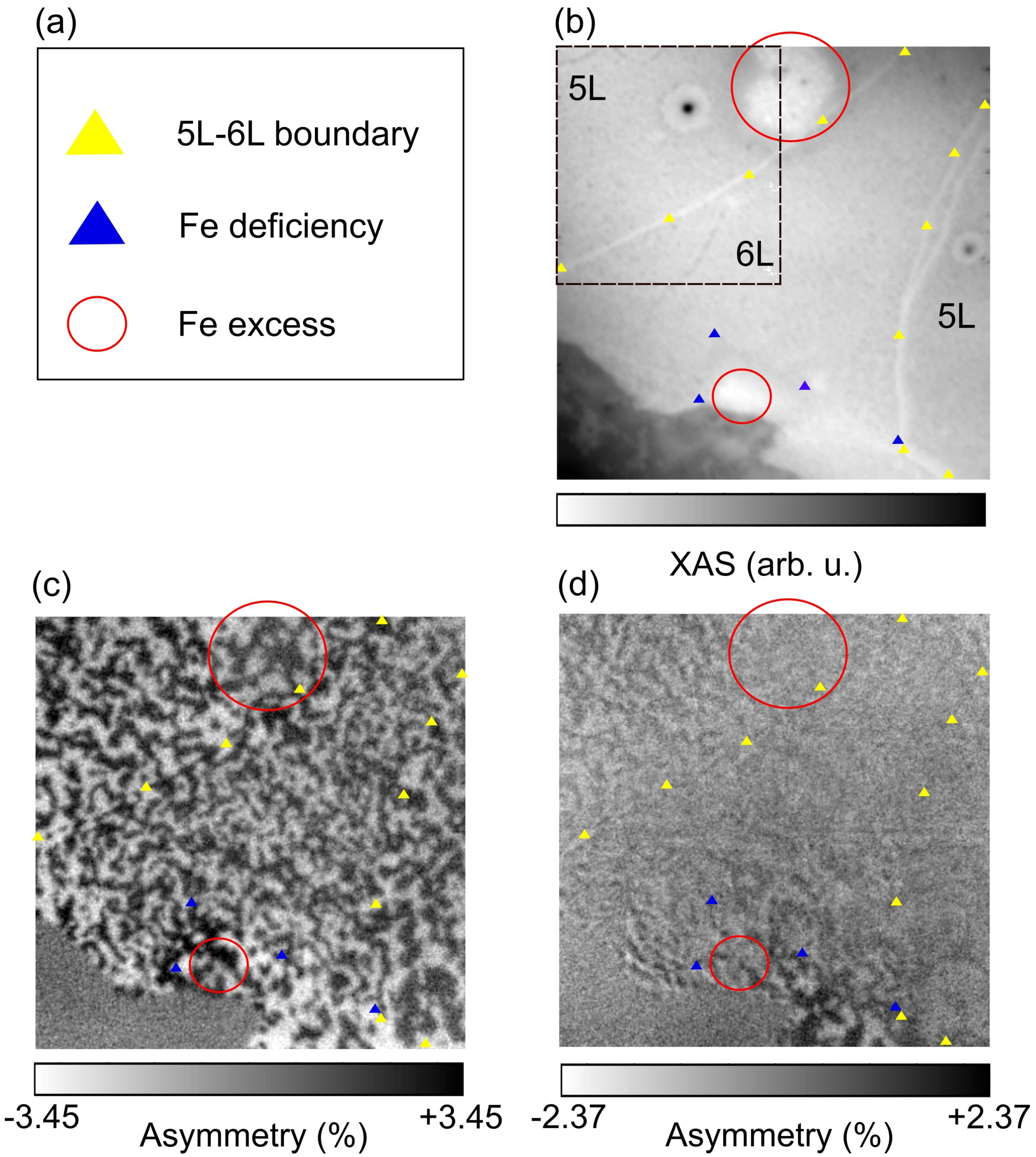

Figure S6: Study of the out-of-plane and in-plane magnetic contrast of 5L and 6L flakes, and its dependence on Fe stoichiometry. (a) The legend denotes the following features: 5L-6L boundaries (yellow triangles), and areas of Fe deficiency (blue triangles) and Fe excess (red circles). (b) XAS and (c) out-of-plane and (d) in-plane magnetic XPEEM contrast, respectively, of 5L and 6L flakes. The magnetic contrast (for both the out-of-plane and in-plane component) is stronger in Fe-deficient areas, while excess Fe leads to a weakening of the magnetic contrast. All images are 10 µm × 10 µm. The dashed black square in (a) indicates the close-up shown in Figure 5 in the main text.

References

(1) Li, Z.; Xia, W.; Su, H.; Yu, Z.; Fu, Y.; Chen, L.; Wang, X.; Yu, N.; Zou, Z.; Guo, Y. Magnetic critical behavior of the van der Waals Fe_5GeTe_2 crystal with near room temperature ferromagnetism. *Sci. Rep.* **2020**, *10*, 15345.

(2) Huang, Y. et al. Universal mechanical exfoliation of large-area 2D crystals. *Nat. Commun.* **2020**, *11*, 2453.

(3) May, A. F.; Ovchinnikov, D.; Zheng, Q.; Hermann, R.; Calder, S.; Huang, B.; Fei, Z.; Liu, Y.; Xu, X.; McGuire, M. A. Ferromagnetism near room temperature in the cleavable van der Waals crystal Fe_5GeTe_2. *ACS Nano* **2019**, *13*, 4436–4442.

(4) Thole, B. T.; van der Laan, G. Branching ratio in x-ray absorption spectroscopy. *Phys. Rev. B* **1988**, *38*, 3158–3171.

(5) Park, T.-E. et al. Néel-type skyrmions and their current-induced motion in van der Waals ferromagnet-based heterostructures. *Phys. Rev. B* **2021**, *103*, 104410.

(6) Stahl, J.; Shlaen, E.; Johrendt, D. The van der Waals ferromagnets $Fe_{5-\delta}GeTe_2$ and $Fe_{5-\delta-x}Ni_xGeTe_2$ — Crystal structure, stacking faults, and magnetic properties. *Z. Anorg. Allg. Chem.* **2018**, *644*, 1923–1929.
